# Better or worse? The prognostic role of the mesenchymal subtype in patients with high‐grade serous ovarian carcinoma: A systematic review and meta‐analysis

**DOI:** 10.1002/cam4.4752

**Published:** 2022-04-17

**Authors:** Juan Chen, Xiaoyan Shi, Lan Xiao, Zelian Li, Zhimin Li, Lei Sun

**Affiliations:** ^1^ Department of Obstetrics & Gynecology First Affiliated Hospital of Anhui Medical University Hefei China; ^2^ Central Laboratory, The Central Hospital of Wuhan, Tongji Medical College Huazhong University of Science and Technology Wuhan China; ^3^ Department of Gynecology Guangdong Women and Children Hospital Guangzhou China

**Keywords:** gene signature, mesenchymal subtype, meta‐analysis, ovarian cancer, prognosis, survival

## Abstract

**Background:**

Tumor characteristics can be prognostically relevant in patients with high‐grade serous ovarian carcinoma (HGSOC). This study aimed to determine whether different subtypes of HGSOC, especially the mesenchymal subtype, are associated with overall survival (OS) or progression‐free survival (PFS) in patients with HGSOC.

**Methods:**

PubMed, Embase, and the Cochrane Library were searched for studies published up to September 2020. The eligibility criteria were (1) population: patients with HGSOG with molecular subtyping of their tumor, (2) exposure: mesenchymal subtype, (3) non‐exposure: differentiated, immunoreactive, proliferative, and other non‐mesenchymal subtypes, (4) outcome: survival, with hazard ratios (HRs), and (5) English language.

**Results:**

The mesenchymal subtype showed no statistically significant difference in OS compared with the immunoreactive subtype (HR = 1.47, 95% CI: 0.78–2.78, *p* = 0.238; *I* ^2^ = 81.2%, *p*_heterogeneity_ = 0.005) or all non‐mesenchymal subtypes (HR = 1.65, 95% CI: 0.97–2.80, *p* = 0.063; *I* ^2^ = 79.4%, *p*_heterogeneity_ = 0.008). The mesenchymal subtype showed no statistically significant difference in PFS compared with the immunoreactive subtype (HR = 1.19, 95% CI: 0.71–2.00, *p* = 0.514; *I* ^2^ = 71.6%, *p*_heterogeneity_ = 0.030) but a significant differences was observed when using all non‐mesenchymal subtypes as reference (HR = 1.51, 95% CI: 1.00–2.28, *p* = 0.049). The results were robust according to the sensitivity analyses.

**Conclusions:**

There are no statistically significant differences in OS between the mesenchymal subtype of HGSOC and other subtypes of HGSOC. Because of statistical power, this meta‐analysis cannot conclude about non‐inferiority, and the relationship between the molecular subtypes and HGSOC prognosis remains controversial. Based on one study, the mesenchymal subtype could have a poorer PFS than the non‐mesenchymal subtypes of HGSOC, but this conclusion requires further evidence.

## Graphical abstract

The molecular subtypes and HGSOC prognosis have a certainly relationship, especially mesenchymal subtype of high‐grade serous ovarian carcinoma (HGSOC) are associated with a poor prognosis.

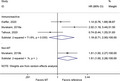

## 1 INTRODUCTION

Ovarian cancer (OC) is an important cause of death in women,^1^, ^2^, ^3^ with a lifetime risk estimated at 1 in 50–70 women.^4^, ^5^, ^6^, ^7^ The peak incidence of OC is observed in women aged 60–64 years,^4^ with the majority of cases of OC being seen in women >50 years.^5^ About 90% of primary OCs are epithelial carcinomas.^5^ Unfortunately, the early diagnosis of OC is difficult, screening is unreliable, and OC symptoms usually do not appear until the advanced stages of the disease.^4^, ^5^, ^6^, ^7^ When present, symptoms often include gastrointestinal complaints and abdominal/pelvic pain.^4^, ^5^, ^6^, ^7^ The 5‐year overall survival (OS) rate is <50% but varies according to disease stage.^7^, ^8^, ^9^, ^10^, ^11^

High‐grade serous ovarian carcinoma (HGSOC) is the most common type of epithelial OC.^12^ HGHSOC has a poor long‐term prognosis because of its late‐stage detection, high metastatic potential, and resistance to cancer drugs.^12^ Recently, large‐scale genomic studies classified HGSOC into molecular subtypes associated with distinct biology and behaviors.^13^, ^14^ The Australian Ovarian Cancer Study (AOCS) identified the molecular subtypes of HGSOC by gene expression analysis (C1, C2, C4, and C5 subtypes).^13^ The Cancer Genome Atlas Research (TCGA) Network study identified four subtypes: immunoreactive, differentiated, proliferative, and mesenchymal.^14^

It is now well recognized that these genomic profiles are associated with OS.^15^ The AOCS revealed that the C5 mesenchymal subtype displays a trend for poorer OS compared with the C2 and C4 subtypes, while the TCGA study showed that survival did not differ significantly among the four subtypes.^13^, ^14^ Hence, the relationship between the molecular subtypes of HGSOC and prognosis remains controversial.

The epithelial‐to‐mesenchymal transition (EMT) is among the initiating events of the metastatic spread of epithelial tumors.^16^, ^17^, ^18^ The genes that reflect EMT are at the core of the mesenchymal gene signature.^19^, ^20^ Therefore, the lack of association between the mesenchymal subtype and poor survival appears paradoxical.^21^ A subsequent re‐analysis of the TCGA dataset revealed that the proliferative and mesenchymal subtypes had the worst OS of all subtypes.^22^, ^23^ Another pooled clustering analysis showed that mesenchymal tumors might have characteristics suggesting less favorable surgical outcomes and poor survival.^24^

Hence, the association between mesenchymal HGSOC and survival remains uncertain. Therefore, we hypothesized that different subtypes, especially the mesenchymal subtype, have a prognostic value in patients with HGSOC, either with OS or progression‐free survival (PFS). We conducted this meta‐analysis to review the literature systematically to test this hypothesis.

## 2 MATERIALS AND METHODS

### 2.1 Literature search

This meta‐analysis was reported according to the Preferred Reporting Items for Systematic Reviews and Meta‐Analyses (PRISMA) guidelines.^25^ Reports were searched for based on the PICO strategy.^26^ PubMed, Embase, and the Cochrane Library were queried using “Ovarian Neoplasms [MeSH]” and relevant keywords for available reports published up to September 2020. Because of the small number of expected studies, the retrieved records were manually screened to avoid missing relevant articles. The eligibility criteria were (1) population: patients with HGSOG with available molecular subtypes, (2) exposure: mesenchymal subtype, (3) non‐exposure: differentiated, immunoreactive, proliferative, and other non‐mesenchymal subtypes, (4) outcome: survival, with hazard ratios (HRs), and (5) English language.

### 2.2 Data extraction

The data were extracted by two different investigators (Juan Chen and Xiaoyan Shi). Study characteristics (authors, year of publication, country, follow‐up time, number of patients or samples, and mean age in each group), treatment parameters (FIGO stage of HGSOC, subtypes based on marker genes, and percentage of residual disease), and primary outcomes (HR of OS or PFS of mesenchymal versus other subtypes, if available) were extracted.

### 2.3 Quality of the evidence

The quality of the articles were assessed independently by two investigators (Juan Chen and Xiaoyan Shi) according to the Newcastle‐Ottawa scale (NOS) for cohort studies.^27^ Discrepancies were resolved through discussion until a consensus was reached.

### 2.4 Data synthesis

The risk estimates were reported as HRs or relative risks (RRs). RRs were analyzed as HRs. Whenever possible, the most adjusted HRs from each study were used in the meta‐analysis.

### 2.5 Statistical analysis

All meta‐analyses were carried out using STATA SE 14.0 (StataCorp). HRs and their 95% confidence intervals (CIs) were compared. Heterogeneity among studies was evaluated using Cochran's *Q*‐test and the *I* ^2^ index. *I* ^2^ > 50% and *Q*‐test *p* < 0.10 indicated high heterogeneity, and the random‐effects model was used; otherwise, the fixed‐effects model was used. *p*‐values <0.05 were considered statistically significant. Publication bias was not assessed using funnel plots and Egger's test because the numbers of studies included in each quantitative analysis were <10, leading to a high risk of incorrect results.^28^, ^29^

## 3 RESULTS

### 3.1 Study selection and characteristics

**FIGURE 1:**
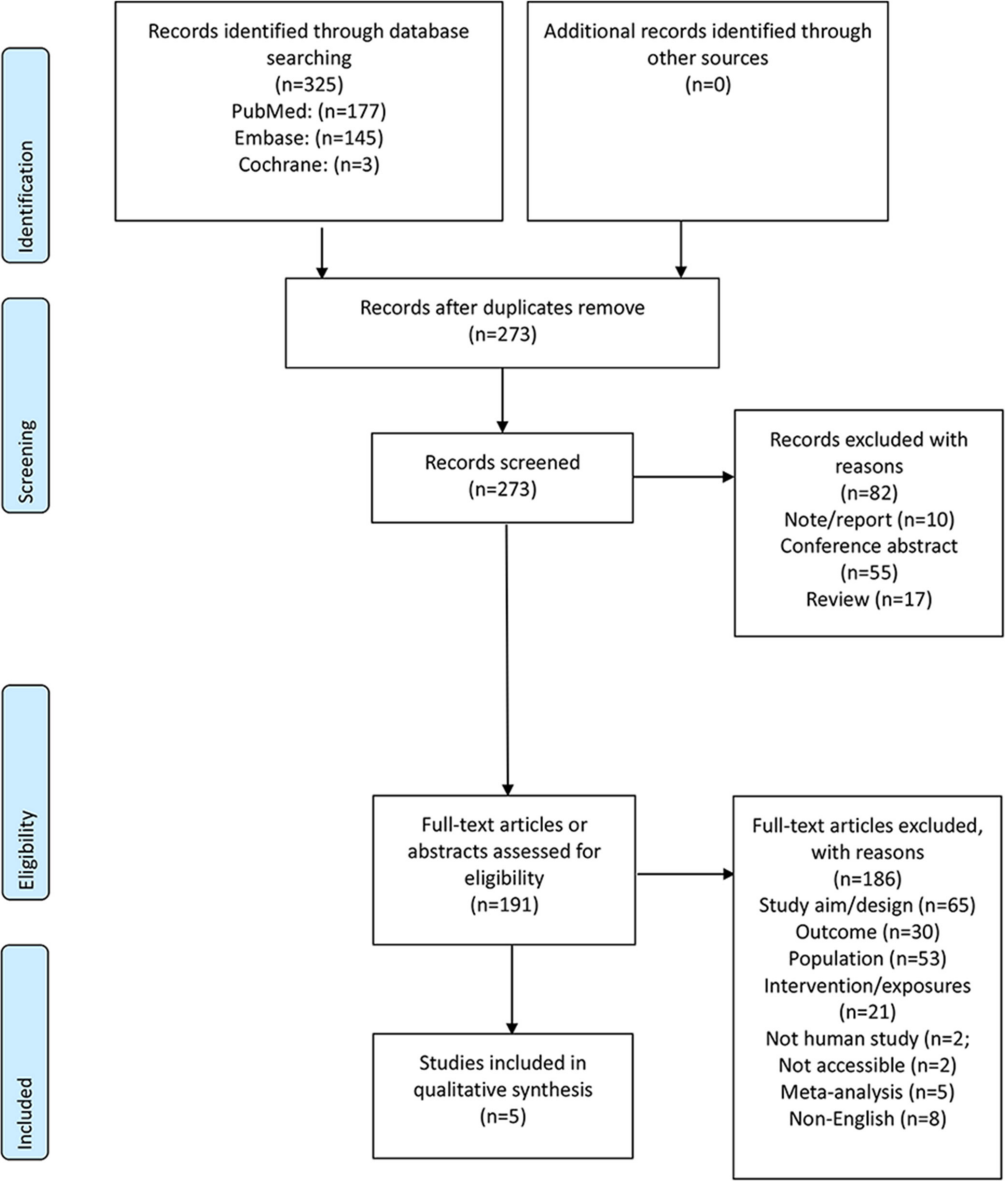
PRISMA 2009 flow diagram

Figure 1 shows the study retrieval process. The search yielded 325 records. After removing the duplicates, 273 records were screened, and 82 were excluded. Then, 191 articles/abstracts were assessed for eligibility, and 186 were excluded (study aim/design, *n* = 65; outcome, *n* = 30; population, *n* = 53; intervention/exposures, *n* = 21; non‐human study, *n* = 2; not accessible, *n* = 2; meta‐analysis, *n* = 5, and non‐English, *n* = 8).

Finally, five studies (1829 patients/studies) were included^30^, ^31^, ^32^, ^33^, ^34^ (Table 1). Two studies were from Japan,^31^, ^32^ one from Europe,^30^ and two from North America.^33^, ^34^ Table S1 shows that three studies^30^, ^31^, ^32^ scored eight stars on the NOS, and two^33^, ^34^ scored nine stars.

**TABLE 1:** Literature search and study characteristic

Author, year	Country	Study design	*n*	Stage	Age (years)	Subtypes based on marker genes, *n* (%)	Residual disease (%)	Follow‐up
Kieffer, 2020^30^	France	Retrospective cohort study	949	FIGO I–IV	AOCS cohort: 59 ± 14.5 Curie cohort: 58 ± 14 TCGA cohort: 59 ± 15.75	AOCS: NA Curie: Differentiated: 30 (28.0) Immunoreactive: 26 (24.3) Mesenchymal: 31 (29.0) Proliferative: 20 (18.7) TCGA: Differentiated: 148 (26.6) Immunoreactive: 129 (23.2) Mesenchymal: 118 (21.2) Proliferative: 138 (24.8)	/	10 years
Murakami, 2016^31^	Japan	Retrospective cohort study	132	FIGO I–IV	M: 59 ± 10.5 IR: 56 ± 10.75 SP: 62 ± 11.75 PG: 61 ± 8	Mesenchymal: 48 (36) Immune reactive: 34 (26) Solid and proliferative: 32 (24) Papilloglandular: 18 (14)	100	150 months
Murakami, 2019^32^	Japan	Retrospective cohort study	201	FIGO II–IV	M: 59 ± 9.5 IR: 57.5 ± 7.5 SP: 59 ± 10.75 PG: 57 ± 13.25	Mesenchymal: 72 (35.8) Immune reactive: 32 (15.9) Solid and proliferative: 49 (24.4) Papilloglandular: 48 (23.9)	100	10 years
Talhouk, 2020^33^	Canada	Retrospective cohort study	213 (samples)	High and low	60.1 ± 10.5	Mesenchymal: 823 (24.3) Immunoreactive: 836 (24.7) Differentiated: 1124 (33.2) Proliferative: 604 (17.8)	61.8	10 years
Torres, 2018^34^	United States	Retrospective cohort study	334	FIGO III–IV	63.5 ± 11.4	Proliferative: 92 (27.5) Differentiated: 73 (21.9) Mesenchymal: 94 (28.1) Immunoreactive: 75 (22.5)	68.7	120 months

Abbreviations: AOCS, Australian Ovarian Cancer Study; FIGO, International Federation of Gynecology and Obstetrics; IR, immune reactive; M, mesenchymal; NA, not applicable; PG, papilloglandular; SP, solid and proliferative; TCGA, The Cancer Genome Atlas.

### 3.2 Overall survival

**FIGURE 2:**
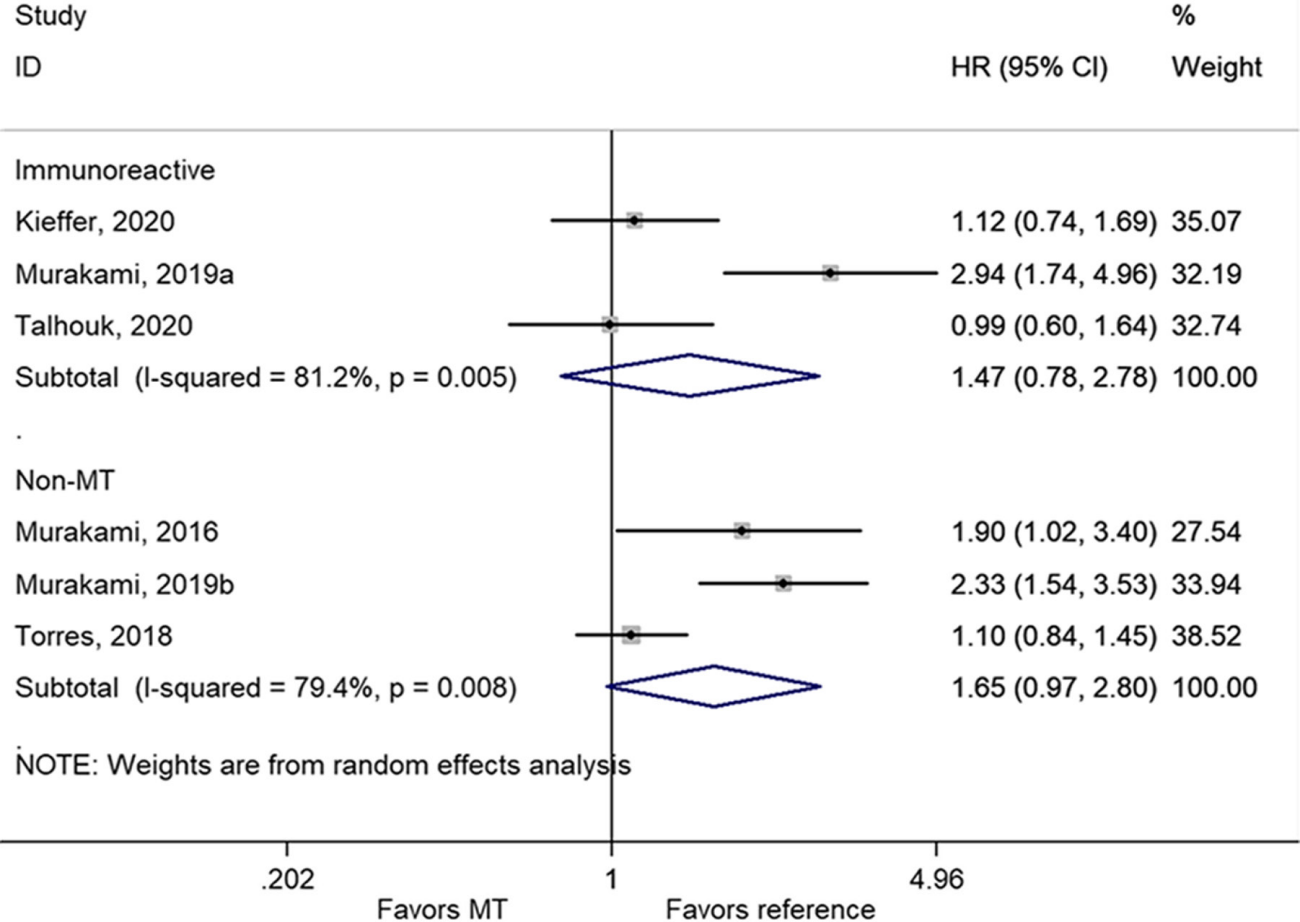
Forest plot of overall survival comparing the mesenchymal subtype with the immunoreactive or non‐mesenchymal subtype

There were no statistically significant differences in OS between the mesenchymal subtype and the immunoreactive subtype^30^, ^32^, ^33^ (HR = 1.47, 95% CI: 0.78–2.78, *p* = 0.238; *I* ^2^ = 81.2%, *p*_heterogeneity_ = 0.005) or all non‐mesenchymal subtypes^31^, ^32^, ^34^ (HR = 1.65, 95% CI: 0.97–2.80, *p* = 0.063; *I* ^2^ = 79.4%, *p*_heterogeneity_ = 0.008) (Figure 2; Table 2).

**TABLE 2:** Results from the meta‐analyses. Mesenchymal subtype versus reference

	*N*	HR (95% CI)	*p*	*I* ^2^ (%)	*p*_heterogeneity_
Overall survival					
Reference					
Immunoreactive	3	1.467 (0.776, 2.776)	0.238	81.2	0.005
Non‐mesenchymal	3	1.650 (0.973, 2.796)	0.063	79.4	0.008
Progression‐free survival					
Reference					
Immunoreactive	3	1.190 (0.706, 2.005)	0.514	71.6	0.030
Non‐mesenchymal	1	1.510 (1.002, 2.275)	0.049	—	—

### 3.3 Progression‐free survival

**FIGURE 3:**
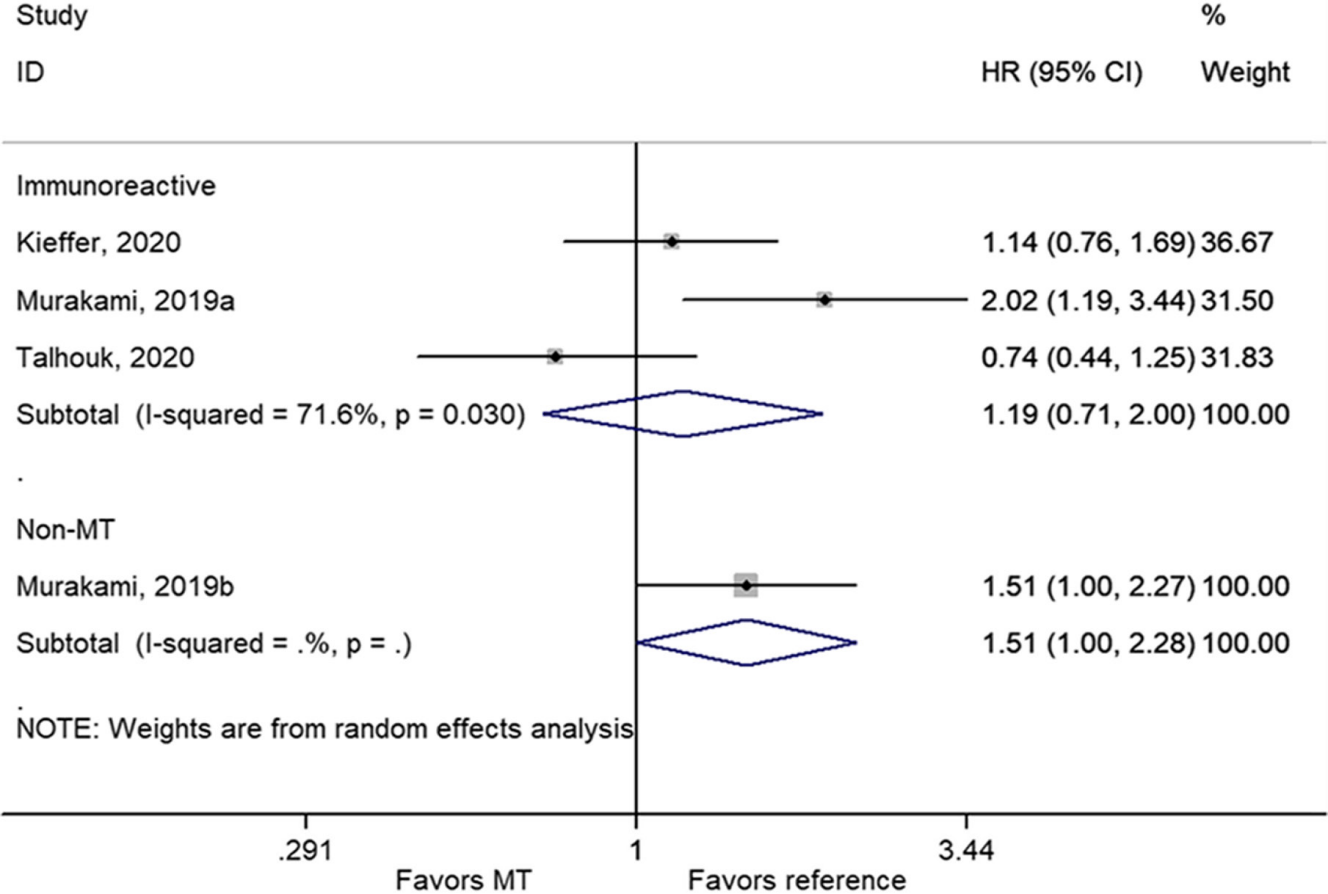
Forest plot of progression‐free survival comparing the mesenchymal subtype with immunoreactive or non‐mesenchymal subtype

There were no statistically significant differences in PFS between the mesenchymal subtype and the immunoreactive subtype^30^, ^32^, ^33^ (HR = 1.19, 95% CI: 0.71–2.00, *p* = 0.514; *I* ^2^ = 71.6%, *p*_heterogeneity_ = 0.030), but there was a significant difference when using all non‐mesenchymal subtypes as reference^32^ (HR = 1.51, 95% CI: 1.00–2.28, *p* = 0.049) (Figure 3; Table 2).

### 3.4 Sensitivity analyses

**FIGURE 4:**
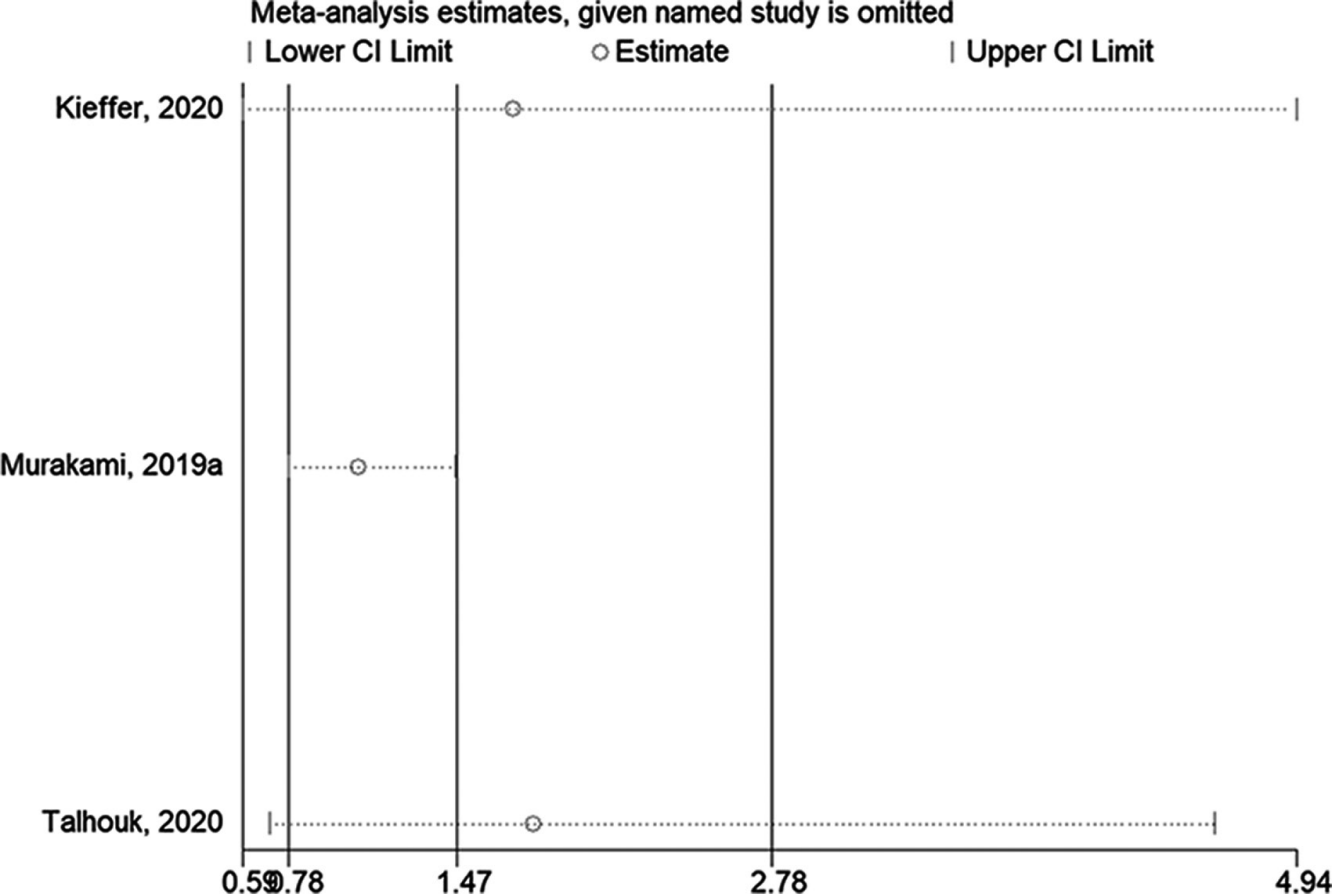
Sensitivity analysis of overall survival comparing the mesenchymal subtype with the immunoreactive subtype

**FIGURE 5:**
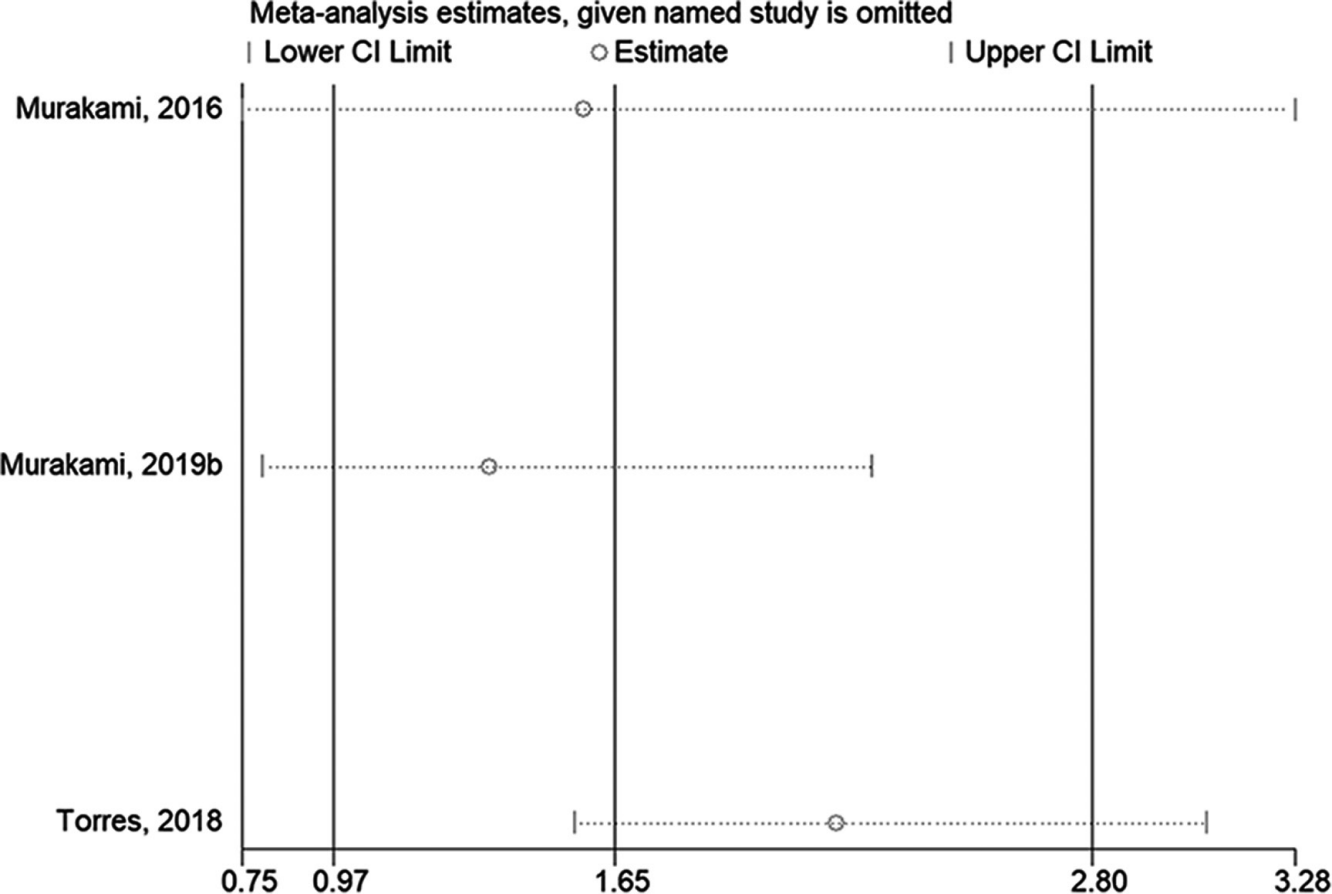
Sensitivity analysis of overall survival comparing the mesenchymal subtype with the non‐mesenchymal subtype

**FIGURE 6:**
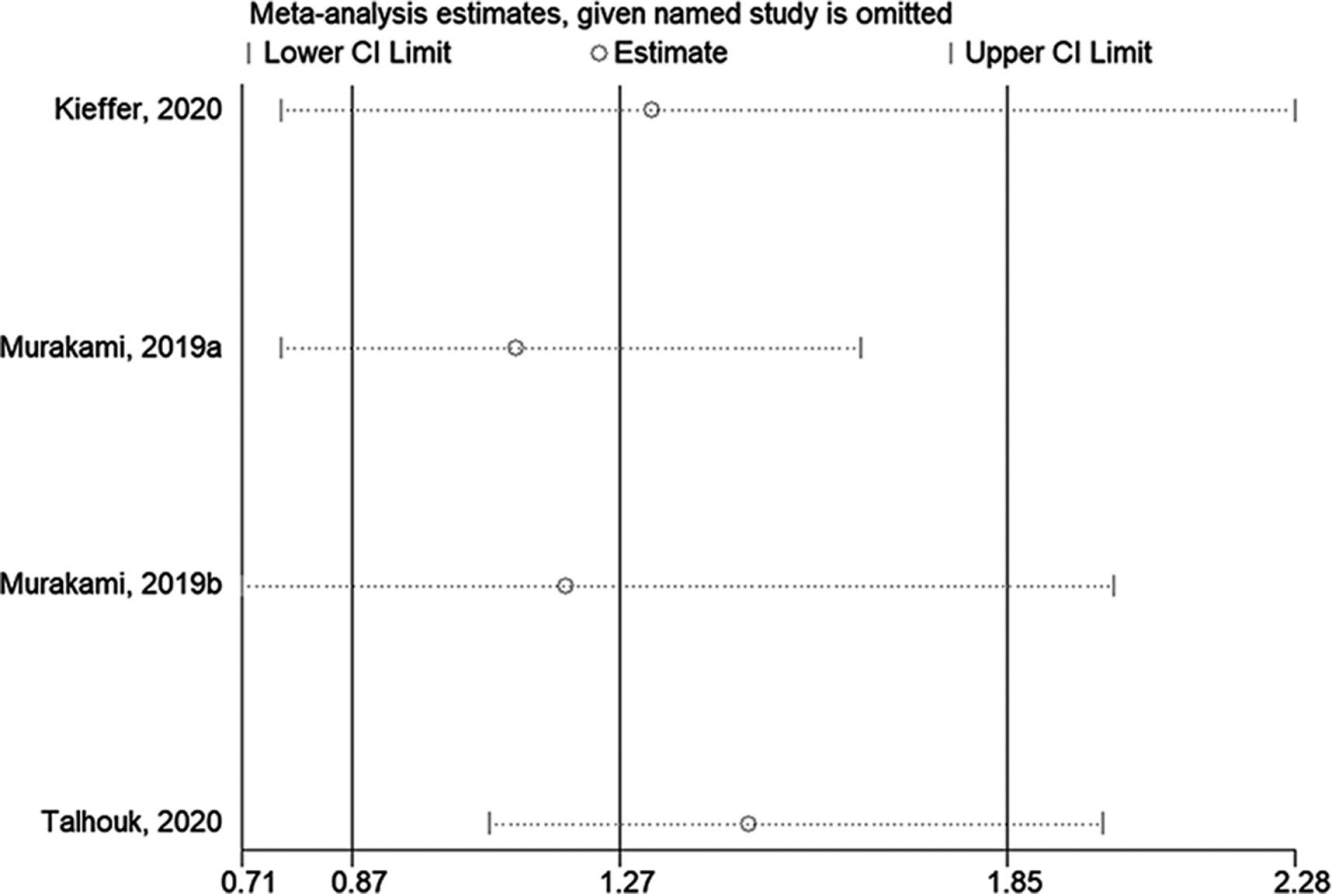
Sensitivity analysis of progression‐free survival comparing the mesenchymal subtype with the immunoreactive or non‐mesenchymal subtype

The sensitivity analysis showed that excluding any one of the studies did not affect the conclusions of OS when comparing the mesenchymal subtype with the immunoreactive subtype^30^, ^32^, ^33^ (Figure 4) or with the non‐mesenchymal subtypes^31^, ^32^, ^34^ (Figure 5). The sensitivity analysis showed that excluding any one of the studies did not affect the conclusions of PFS when comparing the mesenchymal subtype with the immunoreactive or non‐mesenchymal subtypes^30^, ^32^, ^33^ (Figure 6).

## 4 DISCUSSION

The molecular subtypes of HGSOC could be a potential guide for therapeutic decisions. Therefore, this meta‐analysis tested the hypothesis that different subtypes, especially the mesenchymal subtype, are associated with the prognosis (OS and PFS) of HGSOC. The results indicate that there are no statistically significant differences in OS or PFS between the mesenchymal subtype of HGSOC compared with the other subtypes of HGSOC. Because of statistical power, this meta‐analysis cannot conclude about non‐inferiority, and the relationship between the molecular subtypes and HGSOC prognosis is still controversial. Still, the mesenchymal subtype might have a poorer PFS than the non‐mesenchymal subtypes of HGSOC, but this conclusion requires further evidence.

EMT is considered an initiating event in cancer progression by promoting tumorigenesis and metastatic spread.^16^, ^17^, ^18^, ^35^, ^36^ The EMT is also involved in resistance to treatments.^37^, ^38^, ^39^, ^40^ Since the various genes involved in the EMT process are all included in the genomic signatures used to identify the mesenchymal subtype,^13^, ^19^, ^20^, ^41^ it is intuitive to hypothesize that the mesenchymal subtype is associated with a poor prognosis.^21^ The mesenchymal subtype is characterized by low genomic alterations, expression of EMT transcription factors, decreased epithelial marker expression, increased mesenchymal marker expression, and a different cell type composition.^42^, ^43^ Mesenchymal tumors have a high content of stromal cells, and a high proportion of stromal cells has been correlated with a poor prognosis.^43^

The AOCS dataset suggested a poor OS of the C5 (mesenchymal) subtype versus the C2 and C4 subtypes in the AOCS dataset, but the TCGA dataset suggested no associations.^13^, ^14^ Still, more recent re‐analyses of the TCGA dataset revealed a worse OS for the mesenchymal and proliferative subtypes.^22^, ^23^ A review of the genomic classifications of OC suggests that the mesenchymal subtype is associated with a poor prognosis.^1^ A recent multi‐omics study suggested that HGSOCs could be classified into two subtypes: mesenchymal and HRR‐activated.^42^ The patients with mesenchymal HGSOC displayed significantly worse survival than patients with the HRR‐activated subtype.^42^ This classification could support the comparison of mesenchymal HGSOC versus non‐mesenchymal HGSOC. Still, the conflicting results reported in the literature needed to be summarized, and no other meta‐analysis is available on this topic. In the present meta‐analysis, there were no statistically significant differences in OS or PFS between the mesenchymal subtype of HGSOC compared with the other subtypes of HGSOC; a significant difference was observed for PFS, but only when using all non‐mesenchymal HGSOCs as a comparator (but this specific analysis included only one study). Therefore, additional studies are necessary to elucidate this point.

This meta‐analysis was carried out on the premise that differences in prognosis between mesenchymal and non‐mesenchymal HGSOC might influence the therapeutic strategy. Still, alternatives to first‐line therapy are lacking in OC management. The current guidelines do not consider the HGSOC subtypes in their recommendations,^5^, ^7^ probably because of the small amount of available evidence and the conflicting results. Future studies should examine whether the HGSOC subtypes influence treatment outcomes. Anti‐EMT therapies could eventually be of use against mesenchymal HGSOC. Phase I and II trials of the anti‐TGFβ antibody fresolimumab have been conducted in various solid tumors.^44^, ^45^, ^46^ In OC cell lines, blocking TGFβ has been shown to reverse the EMT.^47^ In xenograft mouse models, blocking TGFβ increased platinum sensitivity of the tumors.^48^ Sohn et al.^42^ suggested that high‐EMT HGSOCs might benefit from more aggressive management using targeted therapies such as bevacizumab or dose‐dense or extended chemotherapy. Such patients might also benefit from a more intensive follow‐up and surveillance.

This study has limitations. First, it could not conclude on the prognostic effect of the mesenchymal subtype profile in HGSOC because the available studies have conflicting perspectives, and the number of eligible studies were small. Hence, the numbers of included studies and patients were insufficient to overcome these conflicting views. Second, few studies investigated the association between prognosis and the molecular subtypes of HGSOC. Some studies reported the comparison of HR value of OS and PFS between the mesenchymal subtype and other subtypes, while others did not report such comparisons. Furthermore, each study's survival outcomes were reported differently, probably contributing to heterogeneity. Another source of heterogeneity is the differences in treatment and management, which will affect prognosis and survival. Third, all five studies were retrospective studies affected by the inherent biases of retrospective studies. No relevant randomized controlled trials were retrieved using our search strategies, maybe because this research direction is relatively new. Still, these available studies only partially reflect the survival of patients with HGSOC in the real world. Fourth, because of the small number of studies and differences in data reporting, we could not analyze the influence of key clinical covariates on prognosis (e.g., treatments and tumor stage). Finally, most of the included studies were single‐center studies, so the treatment methods of patients with HGSOC might vary. Such heterogeneity probably caused bias in the results of this study.

## 5 CONCLUSION

In conclusion, there are no statistically significant differences in OS or PFS between the mesenchymal subtypes of HGSOC compared with the other subtypes of HGSOC. Because of statistical power, this meta‐analysis cannot conclude about non‐inferiority, and the relationship between the molecular subtypes and HGSOC prognosis is still controversial. Still, the mesenchymal subtype might have a poorer PFS than the non‐mesenchymal subtypes of HGSOC, but this conclusion requires further evidence. For now, the evidence does not support using the mesenchymal subtype of HGSOC as a marker of poor prognosis. This meta‐analysis highlights the need for additional studies on the subject. Future high‐quality studies with a larger number of patients are encouraged.

## CONFLICT OF INTEREST

The authors have no conflict of interest to declare.

## AUTHOR CONTRIBUTIONS

Lan Xiao and Zhimin Li conceived and designed the study. Lei Sun collected the data. Zelian Li performed the analysis. Juan Chen and Xiaoyan Shi prepared and edited the manuscript. All authors read and approved the final manuscript.

## ETHICS STATEMENT

Ethical approval was not sought from an institutional review board or ethics committee prior to commencing this study.

## Supporting information


Table S1.

## Data Availability

All data generated or analyzed during this study are included in this article and its Supporting Information files.
