# Supplementary material for: Better or worse? The prognostic role of the mesenchymal subtype in patients with high‐grade serous ovarian carcinoma: A systematic review and meta‐analysis
Source: Cancer Med. 2022 Apr 17;11(20):3761–70. doi: 10.1002/cam4.4752 (PMC9582683; doi:10.1002/cam4.4752)
Supplement: Supplementary file 1 — Table S1. [file CAM4-11-3761-s001.docx]

**Supplementary Table S1.** NOS criteria for quality of cohort study

| Study | Representativeness of the exposed cohort | Selection of the non-exposed cohort | Ascertainment of exposure | Demonstration that outcome of interest was not present at the start of the study | Comparability of cohorts based on the design or analysis | Assessment of outcome | Was follow-up long enough for outcomes to occur | Adequacy of follow up of cohorts | Total quality scores |
| --- | --- | --- | --- | --- | --- | --- | --- | --- | --- |
| Kieffer, 2020(30) | ⭐ | ⭐ | ⭐ | ⭐ | ⭐ | ⭐ | ⭐ | ⭐ | 8 |
| Murakami, 2016(31) | ⭐ | ⭐ | ⭐ | ⭐ | ⭐ | ⭐ | ⭐ | ⭐ | 8 |
| Murakami, 2019(32) | ⭐ | ⭐ | ⭐ | ⭐ | ⭐ | ⭐ | ⭐ | ⭐ | 8 |
| Talhouk, 2020(33) | ⭐ | ⭐ | ⭐ | ⭐ | ⭐⭐ | ⭐ | ⭐ | ⭐ | 9 |
| Torres, 2018(34) | ⭐ | ⭐ | ⭐ | ⭐ | ⭐⭐ | ⭐ | ⭐ | ⭐ | 9 |
